# Editorial: Interaction between endocrine and exocrine pancreas

**DOI:** 10.3389/fendo.2022.967066

**Published:** 2022-07-29

**Authors:** Ling Li, S. J. Pandol

**Affiliations:** ^1^ Department of Endocrinology, Zhongda Hospital, School of Medicine, Southeast University, Nanjing, China; ^2^ Institute of Glucose and Lipid Metabolism, Southeast University, Nanjing, China; ^3^ Department of Clinical Science and Research, Zhongda Hospital, School of Medicine, Southeast University, Nanjing, China; ^4^ Division of Gastroenterology, Department of Medicine, Cedars-Sinai Medical Center, Los Angeles, CA, United States; ^5^ Basic and Translational Pancreatic Research, Cedars-Sinai Medical Center, Los Angeles, CA, United States

**Keywords:** crosstalk, exocrine and endocrine pancreata, post-pancreatitis diabetes mellitus, pancreatic cancer-associated diabetes mellitus, precisional diabetes classification

The exocrine and endocrine pancreata are closely linked anatomically and physiologically, but the interaction mechanisms of these two parts have not been clarified. When it comes to clinical practice, diabetes and exocrine pancreatic disease are risk factors for each other. Various exocrine pancreatic diseases, including pancreatitis, trauma, pancreatectomy, and pancreatic neoplasia, can affect the endocrine function of the pancreas and further cause type 3c diabetes mellitus (T3cDM), an endocrine disease (1). T3cDM, also known as diabetes of the exocrine pancreas (DEP), is frequently misdiagnosed as type 2 diabetes mellitus (T2DM) and is undertreated (2). DEP is associated with significantly worse outcomes compared to T2DM, such as poor control of glucose and unstable glycemia and death (3). DEP has impaired pancreatic exocrine secretion and requires insulin therapy earlier in the disease because of a different pathobiology from type 1 diabetes mellitus (T1DM) and T2DM (4). Therefore, differential diagnoses of DEP and other types of diabetes are important for determining optimal therapeutic regimens. Better ability to correctly diagnose DEP, building risk prediction models for DEP, and developing prevention and treatments based on mechanisms are all greatly needed (5). Furthermore, the identification and validation of potential biomarkers for DEP will contribute to the clarification of the mechanisms underlying the association between the interaction between the endocrine and exocrine pancreas. With the evolution of diagnosis and medical technology, DEP is drawing increasing attention and better estimation of its prevalence. This Research Topic provides an update on different aspects of DEP, which aims to shed light on the disease profile.

For one of the most common types of DEP, post-acute pancreatitis diabetes mellitus (PPDM-A) has many associated risk factors. The original study of Lv et al. showed the prevalence of secondary diabetes in Chinese patients after AP and relating risk factors. The study of the authors comprised 1,804 eligible patients from 6,009 new diagnoses of adult-onset pancreatitis, with a median follow-up of 3.04 (IQR 1.73, 4.47) years. The results showed that independent predictors for developing PPDM-A included stress-related hyperglycemia, hyperlipidemia, non-alcoholic fatty liver disease (NAFLD), and recurrent AP. Patients with PPDM-A are often obese and presented with hyperlipidemia and NAFLD, suggesting that a complex signaling system may exist between the pancreas and liver which may play an important role in keeping glucose homeostasis during and after AP. However, the mechanisms involved require further research. Another example of diabetes related to exocrine pancreatic disorders occurs with pancreatic ductal adenocarcinoma (PDAC). The mechanisms of diabetes with PDAC are poorly understood but this cause should be considered in patients who develop diabetes after age 50 who have a decrease in weight (6). The significant international ongoing effort is focused on developing methods for early diagnosis of PDAC based on onset of diabetes in an older aged patient (7). The endocrine and exocrine pancreata are directly modulated by shared common genes, which may be exploited as potential targets for treatment with various therapeutic benefits. A study from Hu et al. investigated the shared genes and common signatures of T2DM and PC *via* WGCNA. They found that S100A6 was upregulated in both T2DM and PC, and that S100A6 promoted PC cell proliferation, migration, and invasion. Moreover, S100A6 was significantly negatively correlated with immune score and closely associated with poor OS. Therefore, S100A6 was recognized as an immune-related biomarker and potential therapeutic target for patients with PC and T2DM. The results provided insights into the common mechanism of S100A6 in PC and T2DM. S100A16 may serve as a prognostic indicator of PC and T2DM, potentially providing a reference for the early diagnosis of diseases, as well as providing a novel therapeutic target.

The clinical characteristics and disease progression vary considerably, and some individuals cannot be clearly classified. Therefore, differential diagnoses of diabetes are important for determining therapy and should arouse great attention of clinicians (8). The case report by You et al. found a type A insulin resistance syndrome (TAIRS) family with a novel heterozygous missense gene mutation type in China. Whole-exome sequencing revealed that both the patient from the Chinese Han family and the father were identified with insulin receptor exon 19c.3472C>T (p.Arg1158Trp) mutation which was previously reported to accelerate insulin receptor degradation and impaired activation of receptor autophosphorylation for activation. The authors meanwhile observed that the patient and his father exhibited high insulin and C-peptide release after glucose stimulation. These findings indicate that this INSR gene mutation may contribute to the development of diabetes through defects in insulin signaling. Thus, detection of genetic markers may provide an important method for differential diagnoses of diabetes. The original study of Li et al. identified five hub genes, namely, TLR4, ITGAM, ITGB2, PTPRC, and CSF1R, as potential biomarkers for DEP by analyzing microarrays. With this study, the authors concluded that TLR4-mediated macrophage activation plays an important role in the pathogenesis of DEP. However, as reported by the authors, further validation of five pivotal genes as potential biomarkers for DEP in a multicenter, large sample population is needed in the future.

In conclusion, this Research Topic provides encouraging data in understanding the interactions between the endocrine and exocrine pancreas, providing information about the incidence, risk factors, and pathogenesis of DEP. However, how pancreatic injury affects endocrine and exocrine function and how they interact and regulate each other are pressing questions. It is essential to identify novel biomarkers and further generate diagnostic models for the early prevention and precision treatment of DEP. A Chinese expert consensus on diabetes subtyping and diagnoses in 2022 is updated to facilitate the clinical precision medicine. Excitingly, the development of new techniques such as multi-omics, space-time omics, single-cell methods, and diabetes antibody tests is now available to bring about new breakthroughs for the disease. In the future, gene, protein, and metabolic biomarkers, risk predictive models relating to DEP, and the revelation of interaction mechanisms on pancreatic endocrine and exocrine function will provide ample evidence for the precise treatment of disease. In addition, based on the innovative concept of the intersection of medical science and engineering, emerging application as clinical therapeutics is offering the possibility of developing new strategies for DEP.

## Author contributions

All the authors have made a substantial, direct and intellectual contribution to the editorial and approved it for publication.

## Funding

This work was supported in part by National Natural Science Foundation of China (81970717 and 82170845).

## Acknowledgments

We would like to thank all authors of this Research Topic for their excellent contributions. We thank the reviewers for their insightful comments, as well as cooperative partners from Key Laboratory of Digital Technology in Medical Diagnostics of Zhejiang Province for various scientific suggestions. We also acknowledge the Frontiers staff for their support.

## Conflict of interest

The authors declare that the research was conducted in the absence of any commercial or financial relationships that could be construed as a potential conflict of interest.

## Publisher’s note

All claims expressed in this article are solely those of the authors and do not necessarily represent those of their affiliated organizations, or those of the publisher, the editors and the reviewers. Any product that may be evaluated in this article, or claim that may be made by its manufacturer, is not guaranteed or endorsed by the publisher.
